# Outer Membrane Vesicles: Current Status and Future Direction of These Novel Vaccine Adjuvants

**DOI:** 10.3389/fmicb.2018.00783

**Published:** 2018-04-26

**Authors:** Kuang Tan, Ruizhen Li, Xiaotian Huang, Qiong Liu

**Affiliations:** Department of Medical Microbiology, School of Medicine, Nanchang University, Nanchang, China

**Keywords:** outer membrane vesicles (OMVs), adjuvants, immunostimulator, vaccine, mucosal delivery carrier

## Abstract

Adjuvants have been of great interest to vaccine formulation as immune-stimulators. Prior to the recent research in the field of immune stimulation, conventional adjuvants utilized for aluminum-based vaccinations dominated the adjuvant market. However, these conventional adjuvants have demonstrated obvious defects, including poor protective efficiency and potential side effects, which hindered their widespread circulation. Outer membrane vesicles (OMVs) naturally exist in gram-negative bacteria and are capable of engaging innate and adaptive immunity and possess intrinsic adjuvant capacity. They have shown tremendous potential for adjuvant application and have recently been successfully applied in various vaccine platforms. Adjuvants could be highly effective with the introduction of OMVs, providing complete immunity and with the benefits of low toxicity; further, OMVs might also be designed as an advanced mucosal delivery vehicle for use as a vaccine carrier. In this review, we discuss adjuvant development, and provide an overview of novel OMV adjuvants and delivery vehicles. We also suggest future directions for adjuvant research. Overall, we believe that OMV adjuvants would find high value in vaccine formulation in the future.

## Introduction

Vaccine adjuvants, functionally defined as non-specific immune-potentiators that provide signals or activate immune-recognition pathways or both, are capable of sustaining robust immune responses to some bacteria and viruses for long duration (Zinkernagel et al., 1997; Steinman, 2008). Conventional vaccines consist of inactivated or attenuated pathogens, but owing to their potential health hazards and risks of reversion in immune-compromised individuals (Belshe et al., 2004), adjuvants have been widely used in vaccine development (Marrack et al., 2009). Recent directions in sub-unit vaccines have also contributed to weak immunity consistent with a failure to induce an effective immune response (Skeiky et al., 2002). Therefore, adjuvant “help” for vaccine formulation is essential to overcome these weaknesses and generate strong immune protection.

Based on their mechanism of action or physicochemical properties, adjuvants could be divided into three subgroups (Allison and Byars, 1991): (I) active immune-stimulants; (II) carriers, and (III) vehicle adjuvants. For instance, Freund's complete adjuvant (FCA) and Freund's incomplete adjuvant (FIA), which mediate “a depot effect” at the injection site and increase the propagation of immune cells, could enhance the specific immune response (Lascelles et al., 1989; Jones et al., 1990); lipopolysaccharide (LPS) of Gram-negative bacteria, being an immunogenic molecule and a stimulator of Toll-like receptors (TLRs), could activate the host immune system against bacterial carriers (Audibert and Lise, 1993); and the liposome adjuvant could extend the half-life of antigens to ensure a significant antigen uptake ratio to APCs after vaccination, inclusive of adjuvant vehicles (Nakanishi et al., 1997).

Adjuvants play key roles in disease prevention and treatment. First, adjuvants influence the immune-phenotype, allowing the vaccine to produce the most effective modes of immunity for each specific pathogen (Edelman and Tacket, 2009; Schijns and Lavelle, 2011). This included potential Th1-promoting adjuvants in treating cancer since Th1 serotype immunity is critical for controlling viruses (Kennedy and Celis, 2008). Second, adjuvants could prolong the antibody response and reduce the antigen dose of immunization, thereby affecting the duration of immune responses (Gołoś and Lutynska, 2015). Third, many adjuvants have effectively facilitated the uptake by the mucosal epithelia against several infectious agents (Srivastava et al., 2015). Fourth, adjuvants could improve immune efficacy in various populations, especially neonates and geriatrics, owing to increased seroconversion and sero-protection rates (Petrovsky and Aguilar, 2004). These abilities of adjuvants are the reason for the continued interest in adjuvant development for a wide array of vaccine designs.

## Limitation of conventional adjuvants

Incorporation of adjuvants into vaccine formulations have helped in overcoming some of the most pronounced limitations of immunization. However, the potential toxicity and adverse reactions associated with adjuvants have not been completely eliminated (Table 1).

**Table 1:** Comparisons of the major adjuvants.

**Adjuvant**	**The merits**	**The drawbacks**	**Mechanism of adjuvanticity**	**References**
Aluminum-based adjuvant	Cheap and widely circulation	Weekly immunity and potential toxicity	The depot effect	Exley, 2014
CFA	Effective	Side effects	The depot effect	Jackson and Fox, 1995
Adjuvant emulisions	Effective	Incomplete immunity, potential toxicity and side effects	Induction of danger molecules and the depot effect	Mohan et al., 2013
Toxin	Effective and mucosal adjuvant	Side effects	PAMPs recognized by PRR and induction of danger molecules	Lee J. B. et al., 2009; Orozco-Morales et al., 2012
Non-toxin proteins	Effective and safe	Incomplete immunity	The “geographic concept”	Bessler et al., 1997
Liposome adjuvant	Comprehensive immunity and delivery vehicle	Side effects	The depot effect and the “geographic concept”	Nakanishi et al., 1997
Immuno-stimulating complexes	Highly effective and could elicit mucosal immune response	A delayed hypersensitivity	The “geographic concept”	Rimmelzwaan et al., 2001
CpG adjuvant	Strong and complete adjuvanticity	Potential side effects and toxicity	PAMPs recognized by PRR and subsequently trigger an immune response	Bode et al., 2003
Cytokines	Specific immune response	Incomplete immunity	Be naturally adjuvant mediated by inducing an inflammatory response	Taylor, 1995
Polymeric particles	Safety, mucosal delivery vehicle and suitable for DNA vaccine in mice models	Toxic and low effective in humans	The depot effect and these materials remained in the tissues for simultaneously extended time of antigen	Manocha et al., 2005
OMVs	Safe and complete immunity, mucosal adjuvant and delivery vehicle	High cost	The presence of PAMPs; induction of danger molecules and the “geographic concept”	Leo et al., 2015

Aluminum-based adjuvants are the most widely used for both human and veterinary vaccines. As the first excipient that had been approved in the vaccine market (Vogel and Powell, 1995), aluminum has been confirmed safe and is an effective Th2 immunity stimulator for preventing infections, such as HIV and malaria (De Milito et al., 2004; Lindblad, 2004). Nevertheless, there are several important limitations to their induction of Th1 immunity, since Th1 cells also have a critical impact on controlling infections. Further, aluminum is a toxic metal that is utilized in a liquid form in vaccine formulations, with unacceptable side effect when used in very high does (Eldred et al., 2006), including adjuvant arthritis, eosinophilia, sterile abscesses, eosinophilia, neurotoxicity, allergenicity, and myofascial pain (Allison and Byars, 1991; Exley, 2014). Finally, poor induction of mucosal immunity also limited its development (Gupta et al., 1995).

CpG adjuvants have been the subject of similar concerns, which led to investigations involving animal models and human subjects. The focus of these studies was safety based on Th1 responses (Bode et al., 2003). However, CpG motifs might reduce the apoptosis of activated lymphocytes, increase the production of auto-antibodies and pro-inflammatory cytokines, and induce TNF-α when administered in a host repeatedly or in conjunction with sub-lethal doses of LPS (Cowdery et al., 1996; Bode et al., 2003; Opal, 2010). This could result in increased host susceptibility to autoimmune disease or a predisposition to toxic shock. Several studies also indicated higher levels of pain, swelling, induration, pruritus, erythema, and systemic symptoms induced by CpG-adjuvanted vaccines (Bode et al., 2003). All these effects elevated uncertainties of potential safety profile of CpG adjuvants.

In general, an ideal adjuvant should be free from unacceptable side effects, and be safe and stable. Its manufacture should be easy, cost effective, and compatible with a wide range of vaccine components (Edelman, 1980; Alaniz et al., 2007). The potential toxicity was the most critical restraining factor to conventional adjuvant development. Additionally, the limited value regarding the minimal stimulation of immunity, led to an understanding and expectation of novel OMV adjuvants, which are safe, induce multifaceted immunity, and suitable for both animals and humans.

## A novel vaccine adjuvant: OMVs

### Structure and function of OMVs

OMVs are ubiquitous in Gram-negative bacteria consisting of proteins; an asymmetric distribution of lipids, mainly as LPS; and periplasmic contents (Kuehn and Kesty, 2005; Ellis and Kuehn, 2010). When bacteria encountered environmental stress, they established a colonization niche that could transport virulent factors and other materials into host cells (McBroom and Kuehn, 2007). Their reaction was an organic defense mechanism that helped pathogens create a suitable micro-environment for biofilm formation, and thus survival in hosts (Schooling and Beveridge, 2006). The effectiveness of OMVs adherence, entry, and content delivery into a host cell cytoplasm were based on the ability of the vesicles to fuse with bacterial membranes (Kulp and Kuehn, 2010). This was supported by endotoxins and lipoproteins in OMVs as conduits for the characteristic transfer of external agents at a sub-cellular level (Aliprantis et al., 2001). The application of vesicle transport has become an engineering tool for the manufacture of vaccines intended for effective antigen delivery.

Additionally, the function of OMVs, which could be used as specific toxin transporters, has proven potency is promoting the immune response, especially in T cell immunity. OMVs contain complex compounds that can be recognized by the innate and acquired immune response pathways through presentation of pathogen-associated molecular patterns (PAMPs) on OMVs. PAMPs could bind to pattern recognition receptors (PRRs) of the antigen presenting cells (ACPs) and activate naïve T cells, thereby activating the immune system (Miyaji et al., 2011). OMVs also possess intrinsic adjuvant properties, which are dependent on their natural composition, including various Toll-like receptor (TLR) antagonists, such as LPS, flagellin, peptidoglycans, lipoproteins, and other outer membrane proteins (OMPs) (Gnopo et al., 2017). Consequently, OMVs have been notable vaccine and vaccine adjuvant candidates for the application of vaccine formulations (Yi et al., 2016).

### The mechanism of OMVs adjuvants

**Figure 1:**
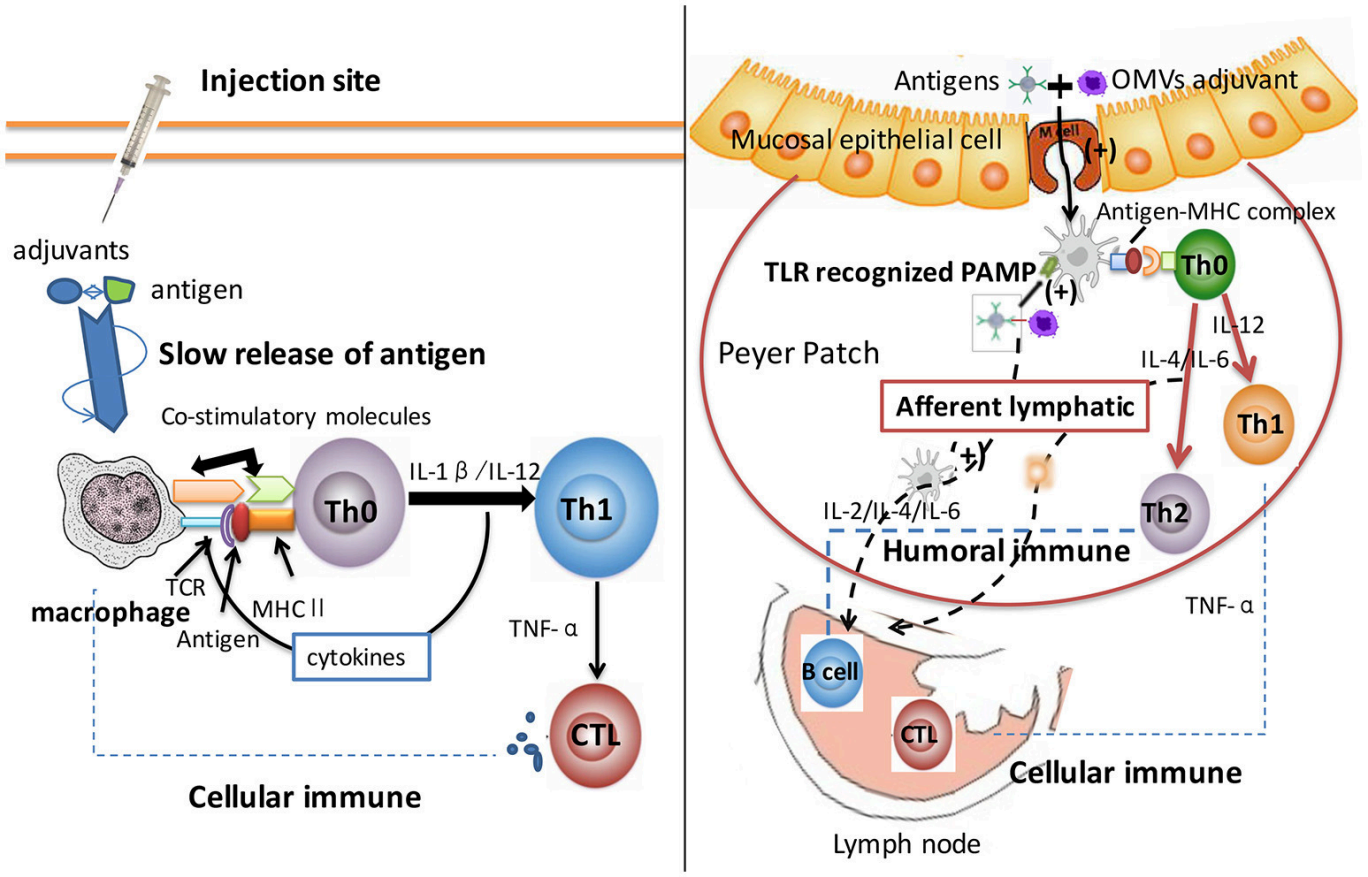
Comparison of aluminum and OMV adjuvants mechanism. After intake by APCs, antigen proteins were processed into smaller components and then loaded onto MHC class II molecules to formulate peptide-MHC complex. Aluminum adjuvant has been proven to induce a “depot effect” with slowly released antigens at the injection site or local lymph node; thus resulting in a prolonged immune response. Aluminum targeted the antigen to APC, and was subsequently recognized by Th0 cell, which had the same peptide-MHC complex recognized receptor. Adjuvants stabilized epitope conformation and stimulated the macrophages to induce retention and activation of Th1 immunity. This progress activated cellular immunity significantly. OMV adjuvant delivered antigen across mucosal barriers, and consequentially enhanced antigen uptake and translocation from the injection site to the tissue-draining lymph node. DCs recognized PAMPs of OMVs that led to the recruitment of immune cells and stimulated APCs through the up-regulated expression of receptors and co-stimulatory molecules. This process enhanced T helper cells production (including Th1 and Th2), and fully amplified cellular and humoral immune systems. The differences of adjuvant mechanisms between aluminum and OMVs caused different types of immune systems. Thus, OMV adjuvant triggered more comprehensive immune response and could serve as novel adjuvants for applications in vaccine development.

Since self-adjuvant properties of OMVs have been independently verifiable, the mechanism of their adjuvant activity could be explained as follows. The basis of OMV adjuvant activity includes the presence of PAMPs, induction of danger molecules, and the “geographic concept” (Figure 1) (Sanders and Feavers, 2011). PAMPs present in OMVs have been recognized by PRRs, mainly TLRs. The activation of TLRs resulted in the recruitment of cells into the immune system to stimulate APCs. This inferred that OMVs could enhance antigen uptake and induce the expression of cell surface molecules, receptors, and co-stimulatory molecules; thus, resulting in enhanced T cells production (Shen et al., 2012). Massari et al. (2006) reported that the binding of *meningococcal* PorB from OMVs to TLR-2 could activate human embryonic kidney cells via theTLR-2/TLR-1 complex, thereby enhancing the serum IgG titer (Massari et al., 2006). Also, some findings suggested that the induction of a danger molecule mediated by damaging host cells could enhance some signal molecules and their causal effect; these activities engaged specific mature T cells resulting in an enhanced immune responses (Siegemund et al., 2007). Furthermore, the “geographic concept” postulates increased uptake and translocation of antigens from the injection site to the tissue-draining lymph node by dendritic cells (DCs). This observation supported the hypothesis that OMVs had great potential for a vaccine platform, since they were immunogenic proteins, delivered carriers, and showed an inherent adjuvant effect.

### Development and advantage of OMVs

OMVs have been tested on animals and humans as vaccine contents for decades, particularly for applications against the disease caused by *Neisseria meningitis* (*N. meningitis*) (Holst et al., 2009). The necessity of this vaccine formulation resulted in a demand of ideal adjuvants for vaccine use. Thus far, many successful experiments have led to the development of OMVs with proven safety and immune stimulating activities that could be developed as adjuvant tools in therapeutic applications. OMVs predisposed as a vehicle and adjuvant for nasal vaccines against *meningococcal* disease were firstly proposed in 1998 (Haneberg et al., 1998). Also, OMVs have been demonstrated compatible with different vaccine platforms (Katial et al., 2002), and were found to stimulate both cellular immunity and humoral immune response, thus possessing comprehensive immune-reactivity (Bottero et al., 2016). OMVs could even function as mucosal transporters to carry antigens to mucosal barriers (Pizza et al., 2001). Hence, OMVs could be an ideal vaccine adjuvant with the capacity for eliciting comprehensive immune responses, superior safety, and formulation of various mucosal vaccines.

Safety is the main concern regarding OMVs in adjuvant development. LPS, as the main structure of OMV, induced immune activity, but also become the main factor affecting the safety; therefore, a low-toxicity OMV structure designed to reduce LPS contents is necessary. LPS naturally resulted in excessive secretion of pro-inflammatory cytokines in organisms (Raetz and Whitfield, 2002); thus, ongoing investigations aimed to discover their compositions and alter their contents to improve OMV safety. Genetic engineering of OMV-producing bacteria has been a valid method to decrease toxicity and facilitate antibody response, and could effectively expand OMV safety in vaccine platforms (Leo et al., 2015). For example, gene knockout experiments conducted by Lee J. B. et al. (2009) depleted the msbB gene of OMVs in *Salmonella typhimurium* to yield low-endotoxic OMVs. This was then fused to the bacterial OmpA protein and constructed into the *Salmonella* mutation, resulting in a significant enhancement of antibody titers in mice serum (Lee S. R. et al., 2009). Also, Kim et al. (2009) established a platform technology by inactivating the MsbB (LpxM) lipid A acyltransferase; thus, generating low toxicity OMVs of *Escherichia coli* (*E. coli*) (Kim et al., 2009). These modified OMVs had both low toxicity and a foreign epitope tag that were suitable for development of multifunctional vaccine delivery vehicles. Additionally, another conservative strategy involved treating OMVs with detergent or detergent-free cell disruption techniques, which was most commonly used with sodium deoxycholate in conjunction with EDTA. The physical or chemical extraction of OMVs could selectively reduce the LPS content and also improving OMV yields (Quakyi et al., 1997).

Mucosal vaccines have been a highly beneficial strategy for preventing a majority of infectious pathogens, since mucosal surfaces are a major entry portal of many pathogenic microorganisms (Neutra et al., 1996; Sardiñas et al., 2006). Adjuvants or delivery carriers for developing an effective mucosal vaccine are essential, since pathogenic antigens alone have not been sufficient for the optimal mucosal delivery of antigens (Srivastava et al., 2015). Nevertheless, most existing adjuvants failed to deliver antigens or effectively activate mucosal immune cells, whereas OMVs-based adjuvants overcame these limitations akin to their complex compositions and functions. Casella and Mitchell (2008) reported that monophosphoryl lipid A derived from *Salmonella* R595, might be a promising mucosal adjuvant (Casella and Mitchell, 2008). Subsequently, Nakao et al. (2011) demonstrated that *Porphyromonas gingivalis* OMVs combined with Poly (I:C) could elicit enhanced secretory IgA (s-IgA) production in mucosal immune response (Nakao et al., 2011). The mechanism of OMV adjuvant processes could describe as follows: OMV is internalized into epithelial cells, when mediated though a lipid-raft-dependent endocytic pathway, and could be directed to early endosome for sorting into lysosomal compartments (Furuta et al., 2009). Effective OMVs could then release antigens recognized and processed by APCs (Ip et al., 2004). PAMPs could also attach to this conditional process for inherent presentation in the outer membrane for interaction with receptors existing in ACPs. These multi-level processes became synergistic and stimulated the production of multiple T cells. As a consensus, the induction of optimal mucosal s-IgA responses to major implements correlated with the presence of CD4 cells secreting IL-4 and IL-5 (Xu-Amano et al., 1993). Furthermore, Bergqvist et al. (2010) proposed that LPS of OMVs could also directly activate B lymphocytes and result in T cell—independent antibody production, which was thought to be a sign of mucosal immune response (Bergqvist et al., 2010). Therefore, OMVs were considered to be attractive mucosal adjuvant by inducing the immune response and transporter activity.

### Application of OMVs adjuvant

Although OMVs were discovered more than 50 years ago, together with the licensed OMV vaccine against *N. meningitidis* for humans, relevant research correlated to its adjuvants properties are still new and insufficient (Acevedo et al., 2014). These limited studied encouraged existing studies (Table 2) that helped in the conclusion of the current investigative finding. These conclusive data could support vaccine engineering with the use of OMV adjuvants in animal and human trials in an attempt to control the spread of bacterial and viral infections.

**Table 2:** Overview OMV adjuvant properties research (continue).

**Microgram-derived adjuvants**	**Years**	**Model**	**Antigen**	**Results**	**Administration routes**	**Comments& addition info**	**References**
*N. meningitidis*	1998	Mice	Inactivated influenza virus	Virus presented with OMVs marked augmented in systemic and salivary antibody responses.	IN route	OMVs might be used as a vehicle or mucosal adjuvant for nasal vaccines against other diseases	Haneberg et al., 1998
*N. lactamica* & *N. meningitidis*	2006	Mice	Hepatitis B surface antigen	IN administration elicited higher IgA response than SC routes, but induced both high IgG response	SC or IN routes	OMV from either *Neisseria* species might act as effective intranasal adjuvants.	Sardiñas et al., 2006
*N. meningitides*	2009	No	No	OMVs stimulated APC overexpress a wide range of co-stimulatory molecules		OMVs might be an active self-adjuvant antigen in vaccine formulation based both on purified proteins on OMVs	Tavano et al., 2009
*E. coli*	2010	Mice	Bacterial protein	Mice inoculated with fusion protein–loaded OMVs had better immunogenic responses than fusion protein alone, antigen alone or empty OMVs		OMVs could serve as vaccines candidate and newer adjuvants for poorly immunogenic antigens	SciBX, 2010
*P. aeruginosa*	2010	Mice	No	Macrophages were more sensitive to OMVs than to pure LPS, flagellin in OMVs could induce inflammatory response.		OMVs of P. *aeruginosa* were potent stimulators of inflammatory responses. And multiple pathogen-specific stimuli were required for maximal immune potency	Ellis et al., 2010
*N. meningitides*	2011	Rabbits	Capsular polysaccharide	OMV induced a high level of bactericidal antibody titer and triggered an opsonophagocytosis activity response	IM routes	OMVs were effective adjuvants but cannot exclude cross-reactivity of protein components in the OMV	Siadat et al., 2011
*E. coil*	2011	Mice	KLH	mOMV significantly enhanced KLH-specific IgG production for T cell priming	IP routes	mOMV with strictly penta-acylated LPS was a safe vaccine adjuvant and could be used in vaccine development against viral diseases and cancer	Dong et al., 2011
*N. meningitides*	2011	Mice	VLPs of HIV	OMV combined with VLP as an immune-potent combination effectively induced IFN-γ and IL-4 production and thus elicited high level of anti-HIV IgG2a production		HIV-1 VLPs combined with *N. meningitides* OMVs seem to be a promising approach in vaccine development against HIV-1.	Aghasadeghi et al., 2011
*N. meningitides*	2013	Mice	PLs and PsA	Recombined meningococcal PLS from serotype A induced IFN-c production, elicited high specific PsA immune responses and a Th1 pattern immune response		OMV could activate cell-mediated immunity and induce a long-term memory response. And also might be extended to other TI-2 antigen	Romeu et al., 2014
*N. meningitides*	2014	Mice	HBsAg	OMV formulated with HBsAg as immune-potent combination significantly elicited high anti-HBsAg IgG, was comparable with the HBsAg +C/IFA regiment.		OMVs were a human-compatible adjuvants, and could be a promising adjuvant in vaccine development against hepatitis B virus.	Sanders and Feavers, 2011
*E. coli*	2016	Mice	AnAPN1, Pfs48/45 and ovalbumin	OMV adjuvants for IN immunization that antibodies and T cell responses against all three antigens could be induced	IN and SC routes	Engineering of OMV could facilitate antigen adherence to mucosal surfaces and boost of the immune response, and thus can apply for vaccination strategy in malaria and other diseases.	Pritsch et al., 2016
*M. tuberculosis*	2016	No	No	*M. tuberculosis* released vesicles were delivery instruments for immunologically active molecules		OMVs of *M. tuberculosis* were important alternative to BCG vaccine to prevent tuberculosis (TB) infection based on a delivery mechanism for immune active molecule	Daliri et al., 2016
*E. coli*	2016	Mice	Bacterial surface glycans	geOMVs successfully displayed S. *pneumoniae* serotype 14 capsule to raise specific antibodies and reduced chicken colonization by C. *jejuni*		geOMVs as vaccines platform could be employed to prevent infections caused by a wide variety of microbial agents in human and animals	Szymanski et al., 2016
*N. meningitidis*	2017	Mice	rPorA	Mice vaccinated with recombinant PorA exhibited a predominant high IgG1 response, increased phagocytic uptake and effective intracellular killing	SC route	Porin A could be a valuable target for the development of immune therapeutic strategies against *N. meningitidis*.	Afrough et al., 2017
*E. coli*	2017	Mice	Influenza virus	Modified OMVs-adjuvanted influenza vaccine induced higher humoral and cellular immune than alum, and could elicit cross-protection against heterologous virus challenges		The modified OMVs could be a promising adjuvants for HIN1 influenza vaccine and might be widely applicable to against influenza virus infection	Shim et al., 2017

*Neissria meningitidis* as an invasive human pathogen can progress to sepsis, meningitis, and death. Owing to the significant epidemic harm that led to the progress in updated generation of *meningococcal* OMV vaccines, a consequential experiment associated with the adjuvants properties for *N. meningitidis* OMV-derived particles was also carried out (Stephens and Zimmer, 2002). In a previous report, *N. meningitidis* MenB OMVs were used as an adjuvant with group A *meningococcal* capsular polysaccharide, then this recombinant vaccine was administrated to New Zealand white rabbits to evaluate bactericidal antibody response and opsonophagocytosis activity against serogroup A *meningococci* (Siadat et al., 2011). Unlike most of the classic and introduced adjuvants causing local and systemic hypersensitivity reactions, OMV was a low-toxicity structure and reliable adjuvant with a high potency to induce a typical T cell response (Tavano et al., 2009). Similarly to *N. meningitidis*, OMVs derived from *E. coli* have also been engineered as adjuvants that when fused with a vesicle-localizing protein and immunogenic antigen, could strongly stimulate both cell and humoral immunity, especially mediated IFN-g and IL-17 T cell dependent response production (SciBX, 2010). Hence, OMV-based adjuvants were comparatively superior to aluminum adjuvants only for triggered B cell immunity.

Some valid strategies already existed for the effectiveness of OMV adjuvants formulated with virus or tumor vaccines. Among them, studies involved in HIV were the most comprehensive. OMV-based adjuvants could effectively enhance induction of IFN-γand IL-4 and further promote Th1-oriented responses (Alatrakchi et al., 2002). Further, OMVs combined with Virus-like particles (VLP) as an immune complex significantly induced high anti-HIV IgG production, particularly with IgG2a dominancy. In addition, a study reported that *N. meningitidis* MenB OMV as an adjuvant was promising in AIDs vaccine development (Aghasadeghi et al., 2011). Furthermore, a recent experiment decked OMVs with tumor antigens that elicited protective anti-tumor responses in immune-competent mice. The engineered OMVs offered synergistic protective activity, resulting in OMV platform that was particularly attractive for cancer immunotherapy (Grandi et al., 2017).

Noticeable advances in the process of proteomic and genetic engineering have led to a concern about the development of recombinant vaccines. OMVs have been attractive candidates for recombinant vaccines, since they are novel vaccine delivery platforms that enhanced recombinant engineering (Mohan et al., 2013; Afrough et al., 2017). Since the initial procedure of most biosynthesized bacterial glycans is similar, it was practical to design glycoengineered OMVs (geOMVs) as bacterial vaccine platforms. GeOMVs could display the O-antigen and surface glycans from different bacteria; thus, they could be effectively formulated with vaccines to prevent a wide array of bacterial infections (Szymanski et al., 2016; Valguarnera and Feldman, 2017). Another genetic engineering strategy involved gene-targeting technology. Plasmids could be transported into OMVs and further modify OMV lumen content, including both LPS functions and attenuated toxicity. The newly designed rOMVs could be used as a more skillful immune modulatory system directed to various vaccine platforms, and have been identified as effective in pandemic H1N1 influenza vaccines (Shim et al., 2017; Watkins et al., 2017). Overall, these findings represented a new direction in tailored vaccine design (Turner and Walper, 2017).

### Concerns associated with OMV adjuvants

Recently, both the medical and research communities raised concerns about OMVs as novel adjuvants in controlling diseases, and it would normally led to greater investigative work. However, OMV adjuvant and related vaccine designs nevertheless raised some questions, and research activities in this potential and important field still remain nascent. OMV adjuvant properties should be at the forefront of therapeutic studies in light of their potential to prevent bacterial and viral infections. Research conclusions would possibly support the findings that OMV adjuvants needed a rational design to trigger optimal immune responses. However, owing to the toxicity of wild-type LPS, OMVs needed to be reformulated, together with some TLR antagonists occurring in OMVs, such as flagellin, lipoproteins, and other OMPs, which might cause uncontrolled responses, such as excess inflammation (Arigita et al., 2005; Thompson et al., 2005). Thus, OMV endotoxins should be removed artificially post-production. One example was the removal of Factor H binding protein from the OMPs of *Neisseria* (Jay Lucidarme et al., 2009). Another concern was that LPS-deficient OMVs commonly showed less effective immunogenicity than wild-type OMVs containing wild-type LPS (Gnopo et al., 2017). Therefore, an optimal balance of the appropriate modification of LPS, containing both of low-toxicity and high-immunogenicity, deserved much deeper research.

If OMVs are commercialized as adjuvants, major problems regarding mass production should be resolved. As we know, the formation mechanisms of OMVs are not explicit, and thus consistent production would be difficult (Vipond et al., 2006). For example, during the upstream process (USP) of bacterial pre-culture, an alternative to antifoam was required to scale up the USP fermentation process. Although most antifoams were not compatible with OMV production processes, and their surfactants could affect OMV integrity or interfere with the OMV purification, antifoam was still the standard method to prevent excessive foaming owing to required aeration at higher cell densities. Consequently, alternative techniques for mechanical foam breaking were be considered as part of the scale-up of the fermentation process (Baart et al., 2007; Leo et al., 2015). Moreover, external components, such as temperature, and the absorption of phages, also impacted OMV production (McBroom and Kuehn, 2007; Eddy et al., 2014). Further, oxidative stress caused by cysteine depletion in *N. meningitides*, or sodium carbonate in *Vibiro cholera* directly influenced the recombinant OMVs yield volume (van de Waterbeemd et al., 2013; Altindis et al., 2014). Therefore, it was found to be crucial to improve related production technology and internal environment conditions.

Up to now, few adjuvant candidates have been licensed for human trials as a direct result of the cost-prohibiting large-scale trials (Christensen et al., 2010). Moreover, some findings reported that LPS derivatives had adjuvant activity similar to that of wild-type LPS in mice model, but acquired no efficiency in humans; these species-specific responses were interpreted as differences in the activation and signaling of TLR complexes (Steeghs et al., 2008). These phenomena highlighted the difficulty of using animal models to evaluate the safety platform and protection of OMV candidate adjuvants in human vaccines. Therefore, to solve the challenge of OMVs-based adjuvants for human applications, more human and comparative animal trials are needed. The issue of high cost could be configured under the importance of scientific development and the likelihood of further therapeutic benefits.

### The orientation of OMVs adjuvants

The continuous development of genomics, biochemistry, and nano-biotechnology, as well as the given advantages, have allowed for the successful development of OMV adjuvants as vaccine platforms. Nevertheless, several focuses have converged to engineer adjuvants with enhanced immunogenicity, greater safety, and wider coverage in the future.

First, current adjuvant engineering focused on higher immune potency. This included the incorporation of two or more adjuvants with different mechanisms of action to enhance the potency and type of the immune response to the vaccine antigen (Cooper and Steele, 1991). Added benefits were pursued with a synergistic effect to kill pathogens or deliver antigens. Therefore, future adjuvants offering comprehensive protection should possibly consist of multiple components. The level of protective efficiency also had a direct impact on the cost and utility of the vaccine platform.

Second, mucosal vaccines were geared toward multiple advantages, including convenient administration, non-invasiveness, high-patient compromise, and suitability for large-scale immunization (Srivastava et al., 2015). However, there are still a few traditional mucosal vaccines ready for use in their present form, owing to limitations in engineering newer, safer and more effective mucosal adjuvants (Lycke, 2012; Pritsch et al., 2016). Adjuvant binding with specific ligands could deliver antigens to specialized epithelial micro-fold cells (M cells), which are the most accessible targets for antigen delivered in mucosal lumen. OMVs could capture, adhere and transport microorganisms by endocytosis to underlying lymphoid immune cells, resulting in OMVs a promising mucosal adjuvant directed to mucosal epithelia (Jang et al., 2004). However, most adjuvant designs to date have been directed toward mucosa, without consideration of the particular role of M cell or the differences between various epithelia. Hence, more subtle designs should take into consideration that OMV adjuvants should be specifically tailored to the target mucosal epithelia (Woodrow et al., 2012).

The physicochemical characteristics of carrier components included size, surface chemistry, and hydrophobicity, also had a fatal effect on antigen crossing mucosal barriers (Rajapaksa et al., 2010; Woodrow et al., 2012). These biochemical activities were influenced by OMV types and administration routes. Studies on the transport of synthetic carriers with controlled size and surface chemistry have provided useful insight into the design criteria of mucosal delivery (Lai et al., 2007). Shakweh et al. (2005) reported that rhodamine 6G-labeled PLGA particles ranging in size from 0.3 to 1 μm were applicable when internalized by mucosal Peyer's patches (Shakweh et al., 2005). The differences in various types of OMV and immune routes have been appropriately reviewed by Gnopo et al. (2017). Accordingly, the synthetic stimuli of biochemical factors and their related sizes influenced the subsequent process of antigen delivery. Therefore, appropriate designs of OMVs played an important function in the progress of their transportation.

## Review and conclusion

As a significant component of the constitution of vaccines, adjuvants have been in development for over 80 years (Marrack et al., 2009). Nevertheless, more than a decade ago, it was necessary to modify the formulation and production of adjuvants based on queries about future direction. For example, the cost of developing new adjuvants was considered prohibitive. The needed investment of millions of US dollars for new vaccines was considered a positive return on investment owing to an expanding market for the sale of vaccines. Unfortunately, the same did not hold true for a niche product development, such as adjuvant. This limitation was contrasted to the continuing deficiencies of conventional adjuvants considering their unacceptable side-effects and incomplete immune-reactivity; inclusive of wholesale preclinical experiments that precluded the large-scale use of adjuvants.

However, new discoveries of antigen delivery through the use of OMVs have created a revolution in understanding OMV mechanisms as adjuvants. This was particularly true when OMVs were mediated in the innate immune system. The development of mucosal vaccine delivery systems has engendered new adjuvant research, which was encouraged by new knowledge driving the development of optimal structure—function relationships to produce more effective vaccine adjuvants. Genetic engineering was another fascinating development to modify OMVs for effective future use in vaccine. Although there are still several barriers to the development of OMV adjuvants, such as large-scale clinical and pre-clinical assessments, limited knowledge of OMV manufacturing process, and insufficient investments, there are many noticeable advantages to warrant detailed investigations for the development of OMV adjuvants that have exhibited comprehensive immune potency, especially in T cell immunity, higher safety, wider coverage, and a considerably robust mucosal delivery carrier. This research has also shown the potential of this recombinant vaccine for HIV-1. Compared to conventional adjuvants, OMVs showed evident superiority for future development. The advent of nano-biotechnology, progress of genomics, immunology, microbiology, and vaccine requirements, individually and combined, make it was possible to overcome the aforementioned challenges. Therefore, we consider that the newly discovered form of OMV adjuvants would progressively serve as delivery carrier platform and could be efficiently applied to efficient vaccine development.

## Author contributions

KT and RL wrote the manuscript. QL and XH revised for its integrity and accuracy. QL approved the final version of this manuscript and takes responsibility for its contents.

### Conflict of interest statement

The authors declare that the research was conducted in the absence of any commercial or financial relationships that could be construed as a potential conflict of interest.

## Abbreviations

OMVs: outer membrane vesicles
FCA: Freund's complete adjuvant
FIA: Freund's incomplete adjuvant
APC: antigen-presenting cells
PRR: pattern recognition receptors
TLR: toll-like receptor
PAMP: pathogen-associated molecular patterns
LPS: lipopolysaccharides
OMPs: outer membrane vesicles
*N. meningtidis*: *Neisseria meningitides*
*E. coli*: *Escherichia coli*
geOMVs: glycoengineered OMVs
M cell: microfold cells
DCs: dendritic cells
USP: upstream process
rOMVs: recombinant outer membrane vesicles
KLH: keyhole limpet hemocyanin
PLS: Proteoliposomes
PsA: polysaccharide A
*N. lactamica*: *Neisseria lactamica*
*P*. aerug*inosa*: *Pseudomonas aeruginosa*
*M. tuberculosis*: *Mycobacterium tuberculosis*
HBsAg: Hepatitis B surface antigen
*S. pneumonia*: *Streptococcus pneumonia*
C. jejuni: *Campylobacter jejuni*
rPoA: recombinant Porin A protein
SC routes: Subcutaneous routes
IN routes: intranasal routes
IP routes: Intraperitoneal routes
IM routes: Intramuscularly routes
VLPs: virus-like particles.
